# Gastric Tuberculosis with Outlet Obstruction: A Case Report Presenting with a Mass Lesion

**DOI:** 10.1155/2013/169051

**Published:** 2013-09-03

**Authors:** Ruth Shifa Ecka, Zeeshn Ahamad Wani, Malay Sharma

**Affiliations:** ^1^Department of Pathology, Jaswant Rai Speciality Hospital, Saket, Meerut, Uttar Pradesh 250001, India; ^2^Department of Gastroenterology, Jaswant Rai Speciality Hospital, Saket, Meerut, Uttar Pradesh 250001, India

## Abstract

Tuberculosis is a major health problem worldwide. In India, it is highly endemic. The most common manifestation is a pulmonary disease, but involvement of the gastrointestinal tract is not uncommon with the ileocecal region being the commonest site. Gastric tuberculosis is rare and usually associated with pulmonary tuberculosis or an immunodeficient state. Here, we report a case of gastric tuberculosis presenting as gastric outlet obstruction in an immunocompetent patient without evidence of pulmonary tuberculosis. Biopsy and PCR confirmed the diagnosis, and the patient responded well to standard antitubercular treatment. Though, gastric tuberculosis is rare, it should be considered as a possibility when patients present with gastric outlet obstruction, particularly in endemic areas with tuberculosis.

## 1. Introduction

Tuberculosis of stomach whether of primary or secondary infection is uncommon [1, 2]. It is usually associated with pulmonary tuberculosis or with immunodeficiency state [3]. Clinically, it resembles peptic ulcer disease or malignancy [4, 5]. We report a case of gastric tuberculosis in an immunocompetent patient presenting with gastric outlet obstruction without evidence of pulmonary involvement.

## 2. Case Report

**Figure 1:**
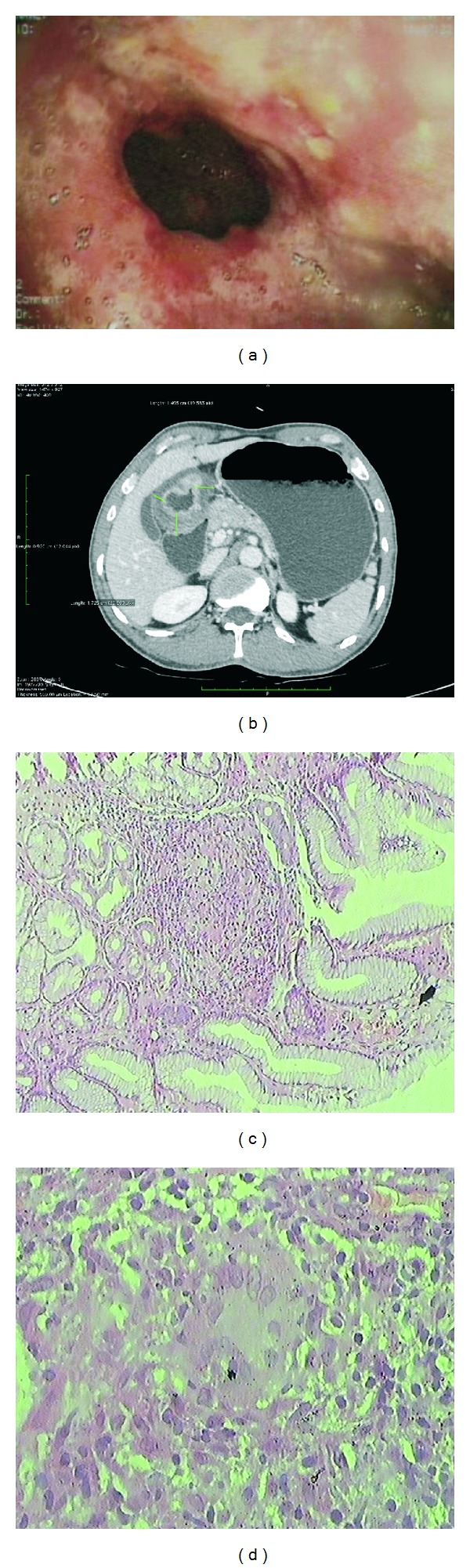
(a) Endoscopic view of the gastric outlet showing an irregular growth with narrowing of lumen. The gastroscope was passed with difficulty beyond this point. (b) Abdominal computed tomography scan showing pyloroantral thickening and distended stomach. (c, d) Histopathological examination image: antral biopsy showing a chronic granulomatous inflammation with Langhans-type giant cells with small foci of caseous necrosis.

A 31-year-old male was referred to our gastroenterology department with a history of recurrent epigastric pain, nausea, and occasional vomiting since 6 months. Epigastric pain was characterized as intermittent, mild, and gnawing in character. The patient also noticed significant weight loss in 2 months. There was no history of fever and no past or family history of tuberculosis. The patient was nondiabetic. A chest X-ray, complete blood count, and liver and renal function tests were normal. ESR was 22 mm/hr. The patient had no known comorbidities and no history of hospitalization. Physical examination only revealed direct tenderness in the epigastrium. There was no lymphadenopathy. His HIV status was negative. Computed tomography scan of abdomen suggested thick antrum with dilated stomach, without any lymph node enlargement or ascites (Figure 1(b)). Upper GI endoscopy showed an irregular mass lesion at the antrum and pylorus region with narrowing of the lumen. (Figure 1(a)). The endoscope was passed beyond this point with difficulty. The impression then was gastric outlet obstruction with enlarged antrum with a possibility of antral malignancy. The patient underwent an endoscopic guided biopsy from the mass lesion. On histopathological examination, multiple epithelioid cell granulomas with Langhans-type giant cells and small foci of caseation necrosis were noted (Figures 1(c) and 1(d)).

As the clinical diagnosis of malignancy was high, the patient was again subjected to 2nd biopsy from multiple sites including a biopsy from the fundus and the duodenum. Tissue for PCR and culture for *Mycobacterium tuberculosis* was also taken in view of an earlier report. Histopathology showed a similar finding, and PCR for *Mycobacterium tuberculosis* was positive. The biopsy from the fundus and the duodenum showed normal morphology. A diagnosis of hypertrophic primary antral tuberculosis was made. The patient was put on antituberculous treatment (ATT) regimen consisting of Isoniazid 5 mg/kg, Rifampicin 10 mg/kg, Ethambutol 15 mg/kg, and Pyrazinamide 25 mg/kg body weight for the initial 2 months followed by Isoniazid and Rifampicin in the same dose for another 7 months. While on treatment, he gained weight and became symptom free. The repeat of the upper GI endoscopy after 4 months of treatment was suggestive of deformed but negotiable antrum. After a followup of 6 months, the patient was symptom free.

## 3. Discussion

Tuberculosis of the gastrointestinal tract most frequently involves the ileocecal region [5]. Involvement of stomach is considered to be rare and is usually secondary to pulmonary tuberculosis [2]. Primary and isolated gastric tuberculosis without evidence of lesions elsewhere is uncommon with only a few cases reported in the literature [1, 4, 6]. The reason for relative rarity is attributed to bactericidal property of gastric acid, scarcity of lymphoid tissue in gastric wall, and the intact gastric mucosa of the stomach. The possible routes of infection include direct infection of the mucosa and hematogenous spread or extension from neighboring tuberculous lesion [5]. The presenting symptoms of gastric tuberculosis are also nonspecific. A review of 23 consecutive cases of gastroduodenal tuberculosis (a 15-year span) in India noted that vomiting (60.8%) and epigastric pain (56.5%) are the most common presenting symptoms. Other symptoms noted are weight loss, upper GI bleeding, and fever with the duration of symptoms varying from 2 days to 15 years [7]. Most reported cases of gastric tuberculosis come from areas with high prevalence of tuberculosis such as India and Africa. Hence, a high index of suspicion is needed when a patient is from a place endemic for tuberculosis. Another emerging concern is the increasing prevalence of human immunodeficiency virus (HIV) infection. The annual risk of developing active tuberculosis when coinfected with HIV is 20–30 times the risk in non-HIV infected individual with an increase in extrapulmonary involvement [3]. Commonly, these patients mimic peptic ulcer disease or malignancy patients, but at times clinical presentation may be misleading. Few cases have unusual presentation as gastrobronchial fistula and massive hematemesis [8, 9]. Our case presented with a mass lesion at the antrum with gastric outlet obstruction, which is the most common presentation of gastric tuberculosis [6, 10, 11]. Tuberculous lesions of the stomach are usually located on the lesser curvature of the antrum and often involve the duodenum with few reports of the involvement of the gastroesophageal junction [10, 12]. It is a well known fact that, probably due to lack of accurate clinical diagnosis, most patients end up with surgical intervention and the diagnosis of gastric tuberculosis is made after surgery. Current guidelines for gastroduodenal tuberculosis suggest that surgery in conjunction with antitubercular therapy (ATT) is the primary therapy. However, the efficacy of endoscopic balloon dilatation along with ATT is also suggested [13]. In our case, only ATT was started as the patient had no acute symptoms. The diagnosis of tuberculosis requires demonstration of caseating epithelioid granuloma or presence of acid-fast bacilli in tissue. When granulomas are noncaseating, small, and discrete, the differential diagnosis on histology includes Crohn's disease, sarcoidosis, syphilis, mycotic lesions, and exposure to beryllium, silicates, or reserpine [5, 10]. Staining for acid-fast bacilli is frequently negative, and the diagnosis is either by culture or by confirming tuberculosis elsewhere [5]. If gastric tuberculosis is clinically suspected, PCR test of the biopsy specimen provides a faster alternative route for the diagnosis while excluding other diagnoses with 100% specificity and 27% to 75% sensitivity [11]. In our case, endoscopic biopsy from pylorus showed caseating granuloma, but acid-fast bacilli was negative, and the diagnosis was confirmed definitely by positive PCR assay and culture. Treatment was successful. Other possibilities of granulomatous lesions were ruled out clinically keeping in mind high incidence of tuberculosis in India. The clinical response to ATT and the repeat of endoscopic examination also supported the diagnosis. 

## 4. Conclusion

Though gastric tuberculosis is rare, in patients presenting with gastric outlet obstruction or with endoscopic evidence of diffuse chronic inflammatory activity, the possibility of gastric tuberculosis should be kept in mind especially in areas endemic for tuberculosis. We summarize that, with the relative rate of extrapulmonary tuberculosis increasing in our country, tuberculosis of the pyloroduodenal area should be considered in the differential diagnosis of gastric outlet obstruction. We also recommend PCR and culture for tuberculosis for such lesions.
